# Factors associated with sexually transmitted infections among sexually active men in Ethiopia. Further analysis of 2016 Ethiopian demographic and health survey data

**DOI:** 10.1371/journal.pone.0232793

**Published:** 2020-05-07

**Authors:** Gizachew Worku Dagnew, Melash Belachew Asresie, Gedefaw Abeje Fekadu

**Affiliations:** Department of Reproductive Health and Population Studies, School of Public Health, College of Medicine and Health Science, Bahir Dar University, Bahir Dar, Ethiopia; University of Pretoria, SOUTH AFRICA

## Abstract

**Background:**

Sexually-transmitted infections are a public health problem in developing countries including Ethiopia. However, there is limited evidence on factors associated with sexually-transmitted infections among men in Ethiopia. Therefore, this analysis was done to fill this gap.

**Methods:**

This analysis was done based on the 2016 Ethiopian demographic health survey data. The survey was a community-based cross-sectional study conducted from January 18 to June 27, 2016. The survey used two stage-stratified cluster sampling technique. A total of 8849 sexually active men were included in this analysis. Descriptive and analytical analyses were performed. A p-value of less than 0.05 was used to declare statistical significance.

**Results:**

Muslim men (AOR = 1.68; 95%CI: 1.02–2.76), men who were not exposed to media (AOR = 1.75; 95%CI: 1.01–3.03) and men who had multiple sexual partners (AOR = 2.29; 95%CI: 1.05–5.01) had higher odds of having a sexually transmitted infection. In addition, men living in Amhara (AOR = 3.31; 95%CI: 1.33–8.22), Oromia (AOR = 4.62; 95%CI: 1.85–11.55), Gambella (AOR = 3.64; 95%CI: 1.27–10.42), and Harari regions (AOR = 4.57; 95%CI: 1.49–14.02) had higher odds of developing sexually transmitted infection. On the other hand, men who believe women are asked to use a condom if she knows he has STIs (AOR = 0.53; 95%CI: 0.33–0.85) had low odds of developing a sexually transmitted infection.

**Conclusions:**

Men not exposed to mass media, Muslims and men with multi-sexual partners had higher odds of having sexually transmitted infections. Encouraging monogamous relationships and exposing men to mass media may help to reduce the burden of STIs in Ethiopia.

## Introduction

Sexually transmitted infections (STIs) are a group of infections for which the primary mode of transmission is through sexual contact. Some of the common STIs other than HIV/AIDS include bacterial vaginosis, herpes, chlamydia, trichomoniasis, gonorrhea, hepatitis B virus and syphilis [1].

Globally, sexually transmitted infections other than HIV, remain a major public health problem. Despite the strong association between STIs and HIV acquisition, STIs other than HIV had been overshadowed in recent years by the heightened public-health focus on HIV treatment. Naturally, STIs affect individuals who are part of partnerships and larger sexual networks and in turn the general populations [2, 3]. The prevalence of STIs remains high although many simple, cheap and cost-effective interventions; as well as prevention strategies, are available to mitigate the transmission [2, 4]. There were an estimated 376 million new curable STIs annually [5–7]. Additionally, more than 500 million people have genital infection with herpes simplex virus (HSV) which predominantly occurred in developing countries [3, 7].

WHO estimated that nearly 1 million people infected every day with any of the four curable STIs: chlamydia, gonorrhea, syphilis, and trichomoniasis with the largest proportion in South and South-east Asia, followed by sub-Saharan Africa, and Latin America [4, 6–8]. In developing countries, STIs and their complications are amongst the top five disease categories for which adults seek health care [9]. In Sab-Saharan Africa, STIs other than HIV are major public health problems. There were more than 93 million annual STI incidence [10].

The incidence, burden, and distribution of STIs in Ethiopia are generally similar to that of other developing countries. According to the Ethiopia demographic and health surveys (EDHS) (2005 and 2011) data, the burden of self-reported abnormal genital discharge increased from 1.4% to 3% among women and 1% to 2% among men from 2005 to 2011. Similarly, genital sore increased from 0.8% to 1% among women and 0.4% to 0.7% among men. These numbers may be underestimated because respondents may be embarrassed or ashamed to report STIs symptoms [11, 12].

Studies showed different levels of STIs (9.4% - 21%) in Ethiopia [13–15]. STIs surveillance study which was conducted in 2013 indicated that vaginal discharge (50%), and urethral discharge (31%) were the two most common syndromes reported. The survey added that 16% of STI patients were co-infected with HIV (8.1% male and 21% female) [16]. In addition to the STIs asymptomatic nature that challenges its prevention and control programs, reports indicate that treatment-seeking for STI is also low. According to the 2011 EDHS report, 63% of women and 56% of men who had an STI or STI symptoms did not seek any advice or treatment; this has a great impact on STIs prevalent in the country [12]. There is also an HIV pandemic that challenges STIs prevention. In 2016, the prevalence of HIV in Ethiopia was 0.9%, of which 1.2 among females and 0.6% among males[17].

Although STIs have public health importance, it remains a neglected area of research. Therefore, this analysis was designed to identify factors associated with STIs among sexually active men in Ethiopia.

## Materials and methods

### Data source

This study was done based on the 2016 EDHS data, collected from January 18 to July 27, 2016. The survey was community-based and cross-sectional, which included 12688 men. The survey used a two-stage stratified cluster sampling technique. The samples were representative at national, regional and residence levels. The survey included all administrative regions and city administrations of Ethiopia. Initially, each region was stratified into urban and rural areas yielding 21 sampling strata. Then, a total of 645 enumeration areas (202 in urban areas and 443 in rural areas) were selected with probability proportional to the enumeration area size. A household listing operation was carried out in all the selected enumeration areas from September to December 2015. Then, 28 households from each cluster were selected using a systematic random sampling technique from the household listing [18].

**Fig 1:**
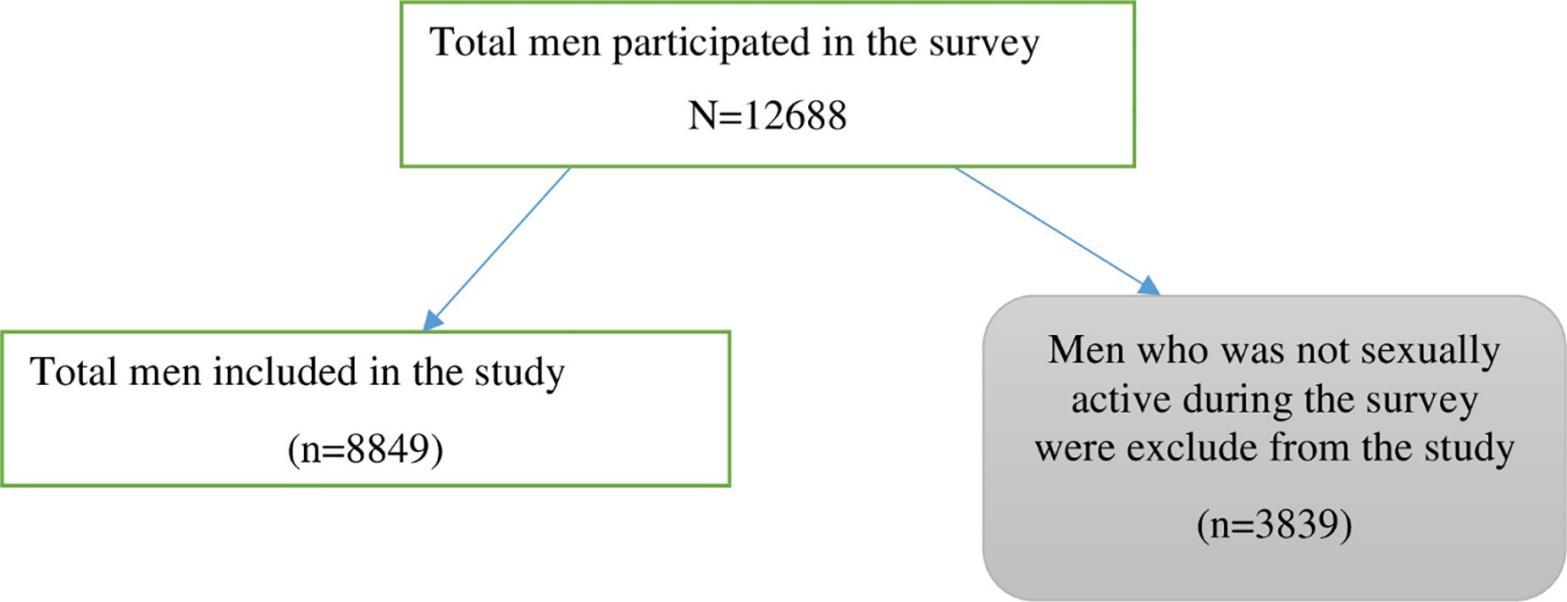
Schematic presentation to select participants to identify factors associated with STI among men Ethiopia; 2016 EDHS.

This analysis included men who have ever had sex. Based on these criteria, a total of 8849 men were included in the final model (Fig 1). In the EDHS, men were asked whether they had an STI sign or symptoms (a bad-smelling, abnormal discharge from the penis or vagina, genital sore or ulcer) in the 12 months before the survey [18].

### Measurements

#### Outcome variable

The outcome variable in this analysis was STIs among sexually active men; a variable with two outcomes (yes/no). It was measured based on the men’s self-report of an STI sign or symptoms 12 months preceding the survey.

#### Independent variables

Potential predictor variables included in this analysis were socio-demographic, sexual behavior and STIs related information and knowledge.

**The socio-demographic variables:** include age, residence, region, religion, education status, marital status, employment status, and wealth-index. The wealth index in the original data set was recoded into three categories as **“**poor” (which included very poor and poor), middle and “rich” (which included rich and very rich in the EDHS data).

**Sexual behavior and practice:** having new sexual partners, number of sexual partners, age at first sexual practice, circumcision, condom use, history of alcohol use and chat chewing. Circumcision variable was coded as not circumcised, circumcised traditionally, and circumcised medically.

**STI knowledge and information;** include mass-media exposure (newspaper/magazine, radio or television), information about STI and knowledge about HIV prevention. Men considered having knowledge about HIV prevention when they responded yes to the questions that consistent condoms use during sexual intercourse and having just one uninfected faithful partner can reduce the chances of getting HIV.

### Statistical analysis

STATA version 14.0 was used to conduct the analysis. Both descriptive and analytical methods of analysis were applied. Descriptive statistics were calculated to characterize men included in the analysis. The data on men were weighted to account for sampling probability and non-response. Besides, the data were adjusted to account for the complex survey design and robust standard errors. Bivariate logistics regression analysis was conducted to select the candidate variable for multivariable logistics regression. Variables with a p-value ≤ 0.2 in the binary logistic regression analysis were taken to the multivariable logistic regression model. Before fitting the final model, multi-collinearity between the independent variable was checked. The multivariable binary logistics regression analysis was done to identify factors associated with STIs. The descriptive results were presented as proportions and the regression results were presented as adjusted odds ratios (AORs) with 95% confidence intervals and p-values. The statistical tests were reported as significant if p-value<0.05 and the 95% confidence interval didn’t contain the null value.

### Ethical approval and consent to participate

The 2016 EDHS protocol was reviewed and approved by the National Ethics Review Committee of the Federal Democratic Republic of Ethiopia, Ministry of Science and Technology and the Institutional Review Board of ICF International. The STATA format data was downloaded from the DHS program with permission.

## Results

### Socio-demographic characteristics of the study participants

More than one-third (36.3%) of men included in the survey were aged ≥ 35 years. About 80% of men were rural residents. About 38% of men involved in the survey did not attend formal education. Regarding marital status, 7462(84.2%) men were married at the time of the survey (Table 1).

**Table 1:** Sociodemographic characteristics of men who were sexual-active, Ethiopia; 2016 DHS.

Variables (n = 8849)	Categories	Frequency (N)	%
Age in years	< = 24	1,117	12.6
	25–29	1,602	18.1
	30–34	2,916	33
	> = 35	3,214	36.3
Residence	Urban	1,741	19.7
	Rural	7,108	80.3
Region	Tigray	531	6.0
	Afar	75	0.9
	Amhara	2,273	25.7
	Oromia	3,339	37.7
	Somali	214	2.4
	Benishangul	100	1.1
	SNNPR	1,743	19.7
	Gambela	29	0.3
	Harari	22	0.2
	Addis Ababa	470	5.3
	Dire Dawa	52	0.6
Wealth index	Poor	3,153	35.6
	Middle	1,663	18.8
	Rich	4,032	45.6
Educational status	No education	3,375	38.1
	Primary	3,558	40.2
	Secondary	1,033	11.7
	Higher	884	10
Marital status	Single	1071	12.1
	Married	7462	84.3
	Others	315	3.6
Religion	Christian	5,879	66.4
	Muslim	2,851	32.2
	Others	119	1.3
Working status	Not working	177	2
	Working for paid in cash	2422	27.4
	Working but not paid in cash	6250	70.6

### Sexual behavior and practice

One-third (33.7%) of men started sex before the age of 19 years. About 57% of participants had ever had a multi-sexual partner. About 95% of men had not used condoms in their most recent sexual intercourse. Of the total men included in the analysis, 3.9% reported that they had paid money for the exchange of sex. Regarding alcohol intake, 488(5.5%) participants reported that they drank alcohol every day. About 72%) participants reported that they had ever heard about STIs. About 70% of men were knowledgeable about HIV prevention. One-third (35%) of the participants reported that they had no exposure to media (Internet, magazine, TV or radio) (Table 2).

**Table 2:** Sexual behavior and practice, and STI related information among sexually active men in Ethiopia, 2016 EDHS.

Variables (n = 8849)	Categories	Frequency (N)	%
Age at first sexual intercourse	< = 18	2979	33.7
	19–24	4449	50.3
	> = 25	1421	16.1
History of multiple sexual behaviors	No	3787	42.8
	Yes	5062	57.2
Number of the sexual partner in the last 12 months	No sexual partner	746	8.4
	one sexual partner	7648	86.4
	multi-sexual partner	454	5.1
Women justified for condom use	No	19.4	1,721
	Yes	80.6	7,128
Condom use during the last sexual intercourse	No	8393	94.8
	Yes	456	5.2
Ever paid someone in exchange for sex	No	8504	96.1
	Yes	345	3.9
Circumcision	No circumcision	600	6.8
	Circumcised traditionally	7,042	79.6
	Circumcised medically	1,206	13.6
Alcohol intake12 preceding the survey	Every day	488	5.5
	At least once a week	2551	25.4
	Less than three a week	1444	16.3
	Not take in the last years	4665	52.7
Frequency of tobacco smoking	Do not smoke	8204	92.7
	Every day	428	4.8
	Some days	217	2.4
Ever heard about STI	No	2463	27.8
	Yes	6386	72.2
Knowledgeable about HIV prevention	No	2684	30.3
	Yes	6165	69.7
Frequency of media exposure	Not at all	3101	35
	less than once a week	2375	26.8
	at least once a week	3373	38.1

### STI prevalence

**Fig 2:**
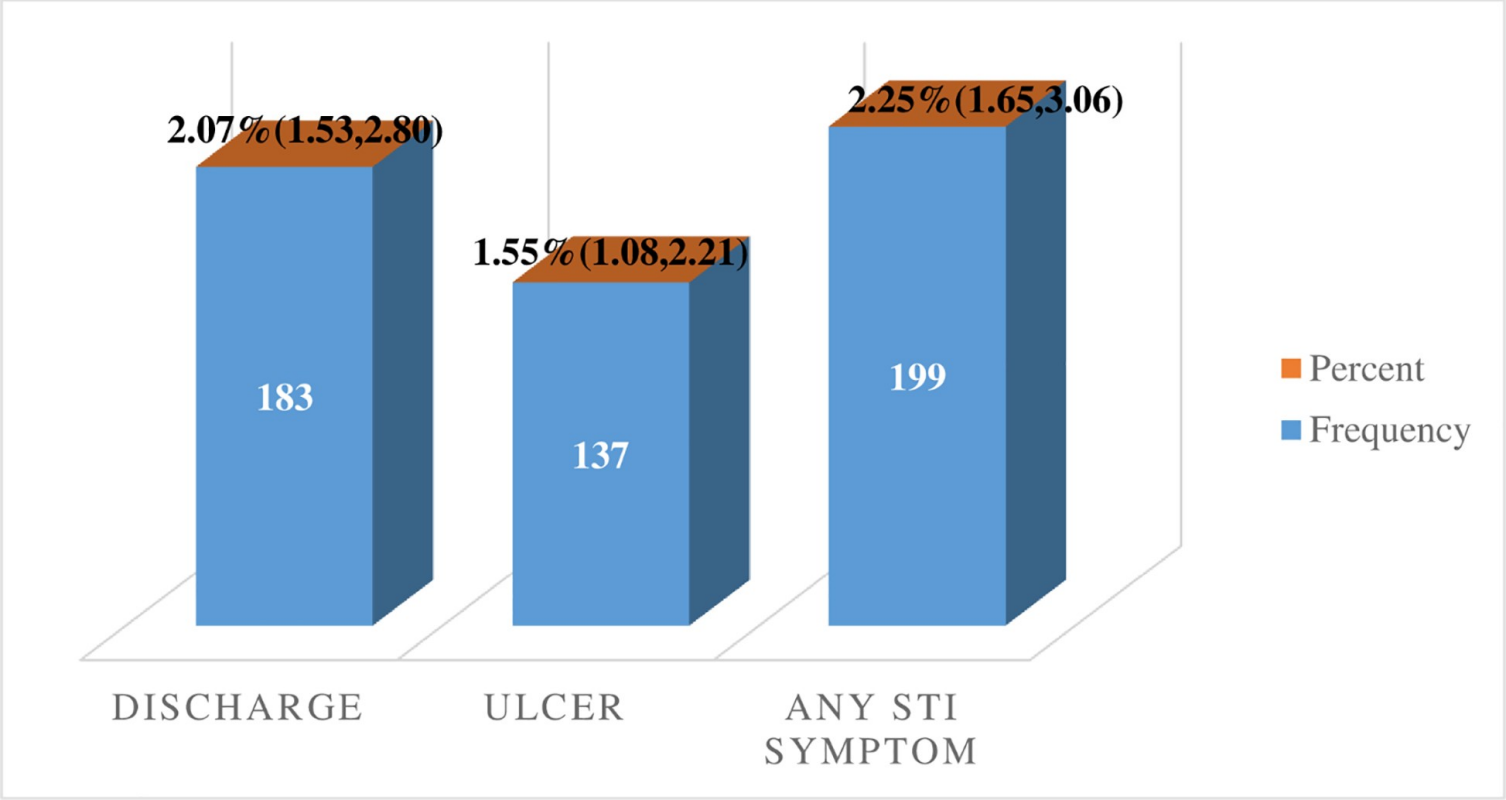
Types of STI syndromes among sexually active men in Ethiopia; 2016 EDHS.

The findings of this study showed that 3.5% (95%CI: 2.8%-4.3%) of the study participants reported they had STIs in the 12 months before the survey More than half (52.3%) of STIs cases had urethral discharge syndrome (Fig 2). Only one-third (35.4%) of men who had STIs or STIs symptoms reported that they sought advice or treatment from a clinic, hospital, private doctor, or other health professionals including from shop/pharmacy.

#### STI prevalence by background characteristics

STI prevalence did not significantly vary by the participants' age, wealth status, and educational level. However, there was a statistically significant variation by region and the number of sexual partners. STI prevalence has had a relative variation across age category at first sexual intercourse (Table 3).

**Table 3:** STI prevalence by selected background characteristics among sexually active men in Ethiopia; 2016 EDHS.

Variables	Categories	Percent with STIs	95% CI
Age	15–24	3.1	(1.8,5.4)
	25–29	3.2	(1.8,5.5)
	30–39	3.9	(2.9,5.3)
	> = 40	3.3	(2.3,4.6)
Wealth index	Poor	3.5	(2.5,5.0)
	Middle	3.5	(2.3,5.4)
	Rich	3.4	(2.6,4.5)
Educational status	No education	3.7	(2.6,5.4)
	Primary	3.3	(2.4,4.5)
	Secondary	3.5	(2.2,5.6)
	Higher	2.9	(1.6,5.3)
Region	Tigray	0.9	(0.4,1.9)
	Afar	2.6	(1.4,4.8)
	Amhara	3.0	(2.2,4.3)
	Oromia	5.6	(4.0,7.7)
	Somali	2.7	(1.4,5.2)
	Benishangul	0.8	(0.4,1.8)
	SNNPR	1.6	(0.9,2.7)
	Gambela	2.9	(1.7,5.0)
	Harari	4.9	(2.6,9.1)
	Addis Ababa	1.5	(0.9,2.6)
	Dire Dawa	2.5	(1.5,4.0)
Frequency of media exposure	Not at all	4.2	(3.0,6.0)
	Less than once a week	3.9	(2.6,5.8)
	At least once a week	2.5	(1.7,3.5)
Age of first sexual intercourse started	< = 18	3.8	(2.8,5.1)
	19–24	3.4	(2.5,4.7)
	> = 25	2.8	(1.8,4.4)
Men beliefs wife justified refusing sex: husband has other women	No	6.3	(4.4,8.9)
	Yes	2.9	(2.3,3.7)

### Factors associated with STI among sexually active men

On multivariable logistics regression, religion, region, the number of sexual partners in the preceding 12 months, belief that women are justified to use a condom if she knows he has STIs and frequency of media exposure were statistically significant at p-value<0.05. Muslim men had higher odds (AOR = 1.68; 95%CI:1.02–2.76) of having STIs compared to Christian men. Participants who live in Amhara, Oromia, Gambella, and Harari regions had higher odds (AOR = 3.31; 95%CI: 1.33–8.22, AOR = 4.62; 95%CI: 1.85–11.55, AOR = 3.64; 95%CI: 1.27–10.42, AOR = 4.57; 95%CI: 1.49–14.02 respectively) of STIs compared to men who lived in Tigray region. Men who had multiple sexual partners had higher odd (AOR = 2.29; 95%CI: 1.05–5.01) of STIs compared to men who had one sexual partner. Men who had not exposed to media had high odds (AOR = 1.75; 95%CI: 1.01–3.03) of STIs compared to those who reported they were exposed at least once a week. Men who believed women are justified to use a condom if she suspected her partner had STIs had low odds (AOR = 0.53; 95%CI: 0.33–0.85) of STIs (Table 4).

**Table 4:** Factors associated with STIs among sexually active men in Ethiopia; 2016 EDHS.

Variables	STI					
	Yes (%)	No (%)	COR	95%CI	AOR	95%CI
**Place of residence**						
Urban	3.6	96.4	1		1	
Rural	2.9	97.1	1.23	0.72–2.08	0.76	0.39–1.48
**Religion**						
Christian	2.4	97.6	1		1	
Muslim	5.4	94.6	2.29***	1.45–3.64	1.68*	1.02–2.76
Others	6.2	93.8	2.65	1.00–7.04	2.54*	1.10–5.83
**Region**						
Tigray	0.9	99.1	1		1	
Afar	2.6	97.4	3.05*	1.09–8.56	1.82	0.60–5.50
Amhara	3	97	3.63**	1.50–8.80	3.31*	1.33–8.22
Oromyia	5.6	94.4	6.80***	2.81–16.48	4.62**	1.85–11.55
Somali	2.7	97.3	3.23*	1.12–9.29	1.51	0.46–4.94
Benishangul	0.8	99.2	0.98	0.32–3.03	0.78	0.24–2.53
SNNPR	1.6	98.4	1.82	0.67–4.92	1.52	0.56–4.18
Gambela	2.9	97.1	3.47*	1.29–9.33	3.64*	1.27–10.42
Harari	4.9	95.1	6.01***	2.12–17.05	4.57**	1.49–14.02
Addis Ababa	1.5	98.5	1.79	0.67–4.79	1.54	0.49–4.79
Dire Dawa	2.5	97.5	2.90*	1.11–7.58	2.05	0.71–5.97
**Circumcision**						
No circumcision	1.6	98.4	0.59	0.22–1.63	0.52	0.18–1.54
Circumcised traditionally	3.7	96.3	1.42	0.79–2.53	1.0 9	0.59–2.00
Circumcised medically	2.7	97.3	1		1	
**Sexual partners in the last 12 months**						
One sexual partner	3.2	96.8	1			1
Multi-sexual partner	6.7	93.3	2.16*	1.06–4.43	2.29*	1.05–5.01
No sexual partner	3.7	96.3	1.15	0.50–2.64	1.27	0.56–2.86
**Women justified using a condom**						
No	5.7	94.3	1		1	
Yes	2.9	97.1	0.49**	0.32–0.74	0.53**	0.33–0.85
**Heard about STI**						
No	2.3	97.7	1		1	
Yes	3.9	96.1	1.71*	1.02–2.85	2.04*	1.16–3.61
**Exposure to media**						
At least once a week	2.5	97.5	1		1	
Less than once a week	3.9	96.1	1.59	0.89–2.85	1.83	1.00–3.36
Not at all	4.2	95.8	1.75*	1.06–2.90	1.75*	1.01–3.03
**Currently, smoke tobacco**						
Do not smokes	3.5	96.5	1		1	
Every day	1.8	98.2	0.51	0.18–1.45	0.38	0.13–1.16
Some days	4.8	95.2	1.38	0.49–3.87	0.97	0.35–2.70

*** p<0.001

** p<0.01

* p<0.05

## Discussion

This study analyzed the national and regional prevalence of STIs among sexually active men in Ethiopia. Muslim men had higher odd of STIs compared to Christian men. This finding was supported by the study conducted in developing countries [19]. The possible reason for this finding might be the difference in marital status. The other reason is that polygamy, which a common risk factor for STI acquisition, is more common among Muslim men in Ethiopia [20–22].

Men who were living in Amhara, Oromia, Gambella, and Harari regions had higher odds of STIs compared to men who were living in the Tigray region. The first reason for this might be a difference in the socio-demographic characteristics of the participants in each region. In this analysis, 4.3% of men from the Tigray region reported that they were Muslim religious followers; which was much lower than the other regions. The second reason might be the difference in men’s attitude on women’s role to prevent STIs. In the current analysis, 94% of women who reside at Tigray region insists their sexual partner to use a condom; this was higher than the Amhara region (87%), Oromia and Gambella (75%) and Harari (64%). The other reason might be the difference in media exposure. In this analysis, 53% of men from the Tigray region reported that they were exposed to mass media at least once a week; which was higher than the other regions. The other reason might also be the difference in regional government commitment to preventing STIs and HIV [17, 23].

Men who reported that they had multiple sexual partners in the 12 months before the survey had high odd of STIs compared to those who reported monogamy relationships. This finding was supported by various studies [13, 24–26]. Having multiple sexual partners is a well-documented risk factor for STI infection. Because of this, abstinence and staying faithful for one sexual partner are the two recommended strategies to prevent STI and HIV globally [3, 16, 27].

Men who believed that a woman is justified in asking her husband to use a condom if she suspects that he has STIs had lower odds of STI compared to their counterparts. The possible explanation for this might be women's negotiation power for practicing safe sex [28]. The other explanation might also be the presence of discussion about STI among men who hold the above opinion, empower them to reduce their risk [29].

The frequency of media exposure had a positive association with STIs among sexually active men. Men who reported that they had no exposure to media had higher odds of STIs compared to those who reported that they had media exposure at least once a week. The possible reason might be the media is broadcasting STI prevention information. The information may have enabled men to avoid unsafe sexual practices to protect themselves from STIs and HIV/AIDS [30].

### Strength and limitations of the study

This analysis identified key factors associated with STI among sexually active men. The study is based on nationally representative data with a large number of participants. The study has a few limitations; this study used secondary data. The analysis did not include important STI risk factors including a partner's sexual behavior. The prevalence of STIs in this study is based on the self-report of STI syndromes. This may underestimate the STI burden due to two reasons. The first reason is that most STIs are asymptomatic. Therefore, men may not report the symptoms. The second reason is that men with STIs may feel embarrassed or ashamed to admit having STIs. Therefore, they may not report the symptoms.

## Conclusions

This analysis revealed that Muslim men, men who live in Amhara, Oromia, Gambela and Harari regional states, men who had multiple sexual partners and men who reported that they were exposed to media at least once a week had had higher odds of STIs. On the other hand, men who believed that women are justified to use a condom if she suspected her partner had STIs had lower odds of STIs. Therefore, health educations interventions focusing on reducing the number of sexual partners are important to reduce STI among men. Besides, increasing media access may reduce STI incidence among men. Special emphasis should be given for Muslim men and men in Amhara, Oromia, Gambella and Harari regions.

## Abbreviations

AOR: Adjusted Odds Ratio
CI: Confidence Interval
EDHS: Ethiopian Demographic and Health Survey
EPHI: Ethiopia Public Health Institute
HIV: Human Immune Deficiency Virus
STIs: Sexual Transmitted Infections
WHO: World Health Organization

## References

[1] Workowski KA. and Bolan GA. Sexually transmitted diseases treatment guidelines, 2015. MMWR. Recommendations and reports: Morbidity and mortality weekly report. Recommendations and reports. 2015 64(RR-03): p. 1 26042815 PMC5885289

[2] Low N., et al Global control of sexually transmitted infections. Lancet. 2006 368(9551): p. 2001–2016. 10.1016/S0140-6736(06)69482-8 17141708

[3] World Health Organization (WHO). Sexually transmitted infections (STIs): the importance of a renewed commitment to STI prevention and control in achieving global sexual and reproductive health. 2012.

[4] World Health Organization (WHO). Global strategy for the prevention and control of sexually transmitted infections: 2006–2015: key messages. Geneva: World Health Organization 2006.

[5] Rowley J., et al Chlamydia, gonorrhea, trichomoniasis and syphilis: global prevalence and incidence estimates, 2016. Bulletin of the World Health Organization. 2019 97(8): p. 548 10.2471/BLT.18.228486 31384073 PMC6653813

[6] World Health Organization(WHO). Report on global sexually transmitted infection surveillance 2018. 2018.

[7] Newman L., et al Global estimates of the prevalence and incidence of four curable sexually transmitted infections in 2012 based on systematic review and global reporting. PloS one. 2015 10(12): p. e0143304 10.1371/journal.pone.0143304 26646541 PMC4672879

[8] Unemo M., et al Sexually transmitted infections: challenges ahead. The Lancet Infectious Diseases. 2017 17(8): p. e235–e279. 10.1016/S1473-3099(17)30310-9 28701272

[9] Aral S.O., Sexually transmitted diseases: magnitude, determinants, and consequences. International Journal of STD & AIDS, 2001 12(4): p. 211–215. 11319969 10.1258/0956462011922814

[10] World Health Organization (WHO). Global incidence and prevalence of selected curable sexually transmitted infections. World Health Organization 2008.

[11] Central Statistical Agency [Ethiopia] and ORC Macro. Ethiopia Demographic and Health Survey 2005. Addis Ababa, Ethiopia, and Calverton, Maryland, USA: Central Statistical Agency and ORC Macro 2006.

[12] Central Statistical Agency (CSA) [Ethiopia] and ICF International. Ethiopia Demographic and Health Survey 2011. Addis Ababa, Ethiopia, and Calverton, Maryland, USA: CSA and ICF International 2012.

[13] Mengistu TS., et al Risks for STIs/HIV infection among Madawalabu University students, Southeast Ethiopia: a cross-sectional study. Reproductive health. 2013 10(1): p. 38. 24069905 10.1186/1742-4755-10-38 PMC3735396

[14] Bereket Yohannes T.G., Mulat Tarekegn. Prevalence and Associated Factors of Sexually Transmitted Infections among Students of Wolaita Sodo University, Southern Ethiopia. International journal of scientific & technology research. 2 2013 2 (2).

[15] Anteneh ZA., Agumas YA., and Tarekegn M. Sexually transmitted diseases among female commercial sex workers in Finote Selam town, northwest Ethiopia: a community-based cross-sectional study. HIV/AIDS (Auckland, NZ). 2017 9: p. 43. 10.2147/HIV.S127319 PMC5339009 28280391

[16] MOH Ethiopia. National Guidelines For The Management Of Sexually Transmitted Infections Using Syndromic Approach. 2015 http://www.moh.gov.et/home.

[17] Kibret G.D., et al, Trends and spatial distributions of HIV prevalence in Ethiopia. Infectious diseases of poverty. 2019 8(1): p. 90 10.1186/s40249-019-0594-9 31623689 PMC6796490

[18] Central Statistical Agency (CSA) [Ethiopia] and ICF. Ethiopia Demographic and Health Survey 2016. Addis Ababa, Ethiopia, and Rockville, Maryland, USA: CSA and ICF 2016.

[19] Drain PK., et al Male circumcision, religion, and infectious diseases: an ecologic analysis of 118 developing countries. BMC Infectious Disease. 2006 6(1): p. 172. 10.1186/1471-2334-6-172 PMC1764746 17137513

[20] Ezra M. Factors associated with marriage and family formation processes in Southern Ethiopia. Journal of Comparative Family Studies. 2003 34(4): p. 509–530.

[21] Hailu A. and Regassa N. Characterization of Women under Polygamous Marital Relationship: (An Examination of Socio-cultural Contexts of Gender Disparities through Qualitative Approach Study in Sidama Zone of Southern Ethiopia). The Oriental Anthropologist. 2008 8(1–2): p. 169–179.

[22] Cook CT. Polygyny: did the Africans get it right? Journal of Black Studies. 2007 38(2): p. 232–250.

[23] EPHI. Ethiopia population-based HIV impact assessment Ephia 2017–2018. December 2018. phia.icap.columbia.edu.

[24] Winston SE., et al Prevalence of sexually transmitted infections including HIV in street-connected adolescents in western Kenya. Sex Transm Infect. 2015 91(5): p. 353–359. 10.1136/sextrans-2014-051797 25714102 PMC4518741

[25] Fenton KA., et al Reported sexually transmitted disease clinic attendance and sexually transmitted infections in Britain: prevalence, risk factors, and proportionate population burden. The Journal of infectious diseases. 2005 191(Supplement_1): p. S127–S138. 15627223 10.1086/425286

[26] Suzanna C. Francis T.N.M., Baisley Kathy, et al Prevalence of sexually transmitted infections among young people in South Africa: A nested survey in a health and demographic surveillance site. PLoSMed, 2018 15(2): p. e1002512. 10.1371/journal.pmed.1002512 PMC5828358 29485985

[27] World Health Organization (WHO). Global strategy for the prevention and control of sexually transmitted infections: 2006–2015: breaking the chain of transmission. 2007.

[28] Wingood GM. and DiClemente RJ. The role of gender relations in HIV prevention research for women. American Journal of Public Health. 1995 85(4): p. 592 10.2105/ajph.85.4.592 7702134 PMC1615134

[29] Leddy A., et al Sexual communication self-efficacy, hegemonic masculine norms and condom use among heterosexual couples in South Africa. AIDS care. 2016 28(2): p. 228–233. 10.1080/09540121.2015.1080792 26344386 PMC4896738

[30] Coyle K., et al Safer choices: reducing teen pregnancy, HIV, and STDs. Public health reports. 2016. 10.1093/phr/116.S1.82 PMC1913682 11889277

